# Chimeric Antibody c.8B6 to O-Acetyl-GD2 Mediates the Same Efficient Anti-Neuroblastoma Effects as Therapeutic ch14.18 Antibody to GD2 without Antibody Induced Allodynia

**DOI:** 10.1371/journal.pone.0087210

**Published:** 2014-02-10

**Authors:** Mickaël Terme, Mylène Dorvillius, Denis Cochonneau, Tanguy Chaumette, Wenhua Xiao, Mitchell B. Diccianni, Jacques Barbet, Alice L. Yu, François Paris, Linda S. Sorkin, Stéphane Birklé

**Affiliations:** 1 ATLAB Pharma, Institut de Recherche en Santé de l’Université de Nantes, Nantes, France; 2 INSERM U.892, Centre de Recherche en Cancérologie de Nantes-Angers, Institut de Recherche en Santé de l’Université de Nantes, Nantes, France; 3 CNRS 6299, Centre de Recherche en Cancérologie de Nantes-Angers, Institut de Recherche en Santé de l’Université de Nantes, Nantes, France; 4 Department of Anesthesia, Mc Gill University, Montreal, Quebec, Canada; 5 Department of Pediatrics, University of California San Diego, La Jolla, California, United States of America; 6 Center of Stem Cell and Translational Cancer Research, Chang Gung Memorial Hospital and Chang Gung University, Taoyuan, Taiwan; 7 Department of Anesthesiology, University of California San Diego School of Medicine, La Jolla, California, United States of America; 8 Université de Nantes, UFR des Sciences Pharmaceutiques et Biologiques, Nantes, France; University of Florida, United States of America

## Abstract

**Background:**

Anti-GD2 antibody is a proven therapy for GD2-postive neuroblastoma. Monoclonal antibodies against GD2, such as chimeric mAb ch14.18, have become benchmarks for neuroblastoma therapies. Pain, however, can limit immunotherapy with anti-GD2 therapeutic antibodies like ch14.18. This adverse effect is attributed to acute inflammation via complement activation on GD2-expressing nerves. Thus, new strategies are needed for the development of treatment intensification strategies to improve the outcome of these patients.

**Methodology/Principal Findings:**

We established the mouse-human chimeric antibody c.8B6 specific to OAcGD2 in order to reduce potential immunogenicity in patients and to fill the need for a selective agent that can kill neuroblastoma cells without inducing adverse neurological side effects caused by anti-GD2 antibody immunotherapy. We further analyzed some of its functional properties compared with anti-GD2 ch14.18 therapeutic antibody. With the exception of allodynic activity, we found that antibody c.8B6 shares the same anti-neuroblastoma attributes as therapeutic ch14.18 anti-GD2 mAb when tested in cell-based assay and *in vivo* in an animal model.

**Conclusion/Significance:**

The absence of OAcGD2 expression on nerve fibers and the lack of allodynic properties of c.8B6–which are believed to play a major role in mediating anti-GD2 mAb dose-limiting side effects–provide an important rationale for the clinical application of c.8B6 in patients with high-risk neuroblastoma.

## Introduction

Neuroblastoma, a cancer derived from precursor cells of the sympathetic nervous system, is the most challenging malignancy of childhood, associated with the highest mortality rate in pediatric oncology; this underlies the need for novel therapeutic approaches [1], [2]. Despite multimodal treatment, the overall survival and event-free survival rates in high-risk patients remain suboptimal. More than half of children diagnosed with high-risk neuroblastoma either do not respond to conventional therapies or relapse after treatment. A major advance in the treatment of these patients was immunotherapy with a chimeric anti-GD2 therapeutic antibody combined with IL-2 and GM-CSF which significantly improved event-free survival and overall survival in a phase III randomized clinical trial in children with high-risk neuroblastoma [3].

Ganglioside GD2, a sialic acid containing glycosphingolipid, is a neuroblastoma-associated antigen. GD2 monoclonal antibodies (mAb) mediate lysis of neuroblastoma cells via complement-dependant cytotoxicity (CDC) and by activation of Fc receptors on immune effector cells resulting in antibody-dependant cellular cytotoxicity (ADCC) [4]. Anti-GD2 antibody infusion is, however, frequently associated with dose limiting severe pain and perceived pain in response to light touch (allodynia) [5], [6]. Moreover some patients have developed sensorimotor polyneuropathy with or without the syndrome of inappropriate antidiuretic hormone subsequent to treatment with anti-GD2 monoclonal antibodies [7]. These adverse effects were attributed to the mAb recognition of GD2 on the pituitary gland and on peripheral nerves followed by complement activation [8]. There is thus an urgent need to develop less toxic anti-GD2 therapeutic mAb.

In an effort to decrease the neurological toxicities of ch14.18, a humanized antibody was recently designed in which the Fc region was mutated in the CH2 domain to no longer engage C1q. The resultant antibody, hu14.18 K322A, retained potent ADCC activity against GD2-expressing tumor with impaired complement activation *in vitro* and reduced allodynia effects in rats [9]. This format would not be suitable, however, for developing more potent immunotherapeutic agents by conjugation of anti-GD2 mAbs to toxins, radionuclides or other effector molecules because it retains its binding activity to peripheral nerve fibers. In another strategy, a pretreatment dose of heat-modified anti-GD2 mouse mAb 3F8 (HM3F8) lacking effector functions was given prior to 3F8, allowing 3F8 dose escalation. It was suggested that HM3F8 might block GD2 on nerves, thereby reducing nerve-related adverse effects of a subsequent dose of native 3F8, and at low dose it would not have any deleterious effect on 3F8 targeting neuroblastoma cells in patients [10].

In our laboratory, we have generated a mouse monoclonal antibody that is specific for the O-acetylated derivative of GD2 (OAcGD2) with no cross-reaction to GD2 observed by immune-TLC [11]. Like GD2, Ganglioside OAcGD2 is expressed highly by GD2-positivetumor cells [12], [13], but importantly it is not found on human peripheral nerve fibers [14]. These properties provide distinct advantage to mAb 8B6 for selectively targeting to neuroblastoma *in vivo* and suggest that anti-OAcGD2 antibodies have the potential to be less toxic than anti-GD2 therapeutic antibodies. Murine mAbs, however, are highly immunogenic in human: they induce Human Anti-Mouse Antibody (HAMA) response in patients, thereby reducing clinical efficacy by removing circulating antibodies [15]. Thus, a mouse/human chimeric version of mAb 8B6 has been made to facilitate clinical development of anti-OAcGD2 mAbs. In the present study, we describe the generation and characterization of a chimeric mouse-human anti-OAcGD2 antibody (c.8B6). The chimeric IgG1 c.8B6 antibody was obtained from the mouse mAb 8B6 to OAcGD2 and expressed in CHO-S cells. It retains the same antigen binding affinity and specificity as its parental mouse mAb. *The in vitro* and *in vivo* antitumor activity of c.8B6 was further examined in the mouse NXS2 neuroblastoma model and compared with ch14.18 anti-GD2 therapeutic antibody.

## Materials and Methods

### Cell Lines

Mouse neuroblastoma Neuro 2a cells and mouse lymphoma YAC-1 cells were obtained from the American Tissue Culture Collection (ATCC). Mouse neuroblastoma NXS2 cell line was given to us by Dr. H. N. Lode (Universitätsklinikum Greifswald, Greifswald, Germany). Human NK-92 cells transfected with CD-16 (RFcγIII) were a gift from Dr. B. Clemenceau (INSERM U. 892, Nantes, France) [16]. CHO-S cells obtained from Life Technologies (Saint-Aubin, France). NXS2 cells were grown at 37°C in 5% CO_2_ in DMEM with 10% heat-inactivated fetal calf serum, 2 mM L-Glutamin, 100 units/mL penicilline, and 100 µg/mL streptomycin. Neuro-2a and YAC-1 cells were grown at 37°C in 5% CO_2_ in RPMI 1640 with 10% heat-inactivated fetal calf serum, 2 mM L-Glutamine, 100 units/mL penicillin, and 100 µg/mL streptomycin. Human NK-92 cells transfected with CD-16 were grown at 37°C in 5% CO_2_ in RPMI 1640 with 10% heat-inactivated fetal calf serum, 2 mM L-Glutamine, 100 units/mL penicillin, 100 µg/mL streptomycin and 100 U/ml human recombinant IL-2. CHO-S cells were grown in suspension at 37°C in PowerCHO 2 serum-free CD medium (Lonza, Basel, Switzerland) supplemented with 4 mM L-Glutamine and ProHT reagent (Lonza).

### Antibodies

Chimeric mAb ch14.18 (γ1,κ) specific for GD2 was kindly provided by Dr. H. N. Lode (Universitätsklinikum Greifswald, Greifswald, Germany). Rituximab®–a chimeric IgG1 mAb against the human protein CD20, which is primarily found on B cells–was used as a negative control. Chimeric anti-OAcGD2 mAb c.8B6 was constructed by joining the complementary deoxyribonucleic acid for the variable region of the murine antibody 8B6 [11] with the human constant regions of the γ1 heavy chain and the κ light chain. The light and heavy chain expression vectors for chimeric antibody were constructed using the same method as described in a previous report [17]. Appropriate light and heavy expression vectors were co-transfected into Chinese hamster ovary (CHO-S) cells and stable transfectants were isolated. The clone producing the highest amount of recombinant mAb was grown in serum-free medium. Monoclonal antibody c.8B6 was affinity-purified from culture supernatant by protein A affinity chromatography followed by anion-exchange chromatography on Sepharose Q for endotoxin removal. The purity of mAb preparations was verified by SDS-PAGE analysis. Endotoxin quantitation was evaluated using the LAL kinetic chromogenic assay (Lonza).

### Flow Cytometry Analysis

Analysis of cell surface OAcGD2- and GD2-expression on tumor cells was performed by indirect immunofluorescence measured by flow cytometry. Briefly, cells were incubated with either mAb c.8B6, ch14.18 or Rituximab at 10 µg/mL for 30 min at 4°C. Antibody binding was detected by incubation with a fluorescein-isothiocyanate-conjugated-labeled F(ab’)_2_ fragment of goat anti-human IgG (Jackson Immunoresearch, Soham, UK) for 30 min at 4°C. Cell fluorescence was analyzed using a FACSCalibur flow cytometer (BD Biosciences, San Jose, CA, USA) and CellQuestPro software (BD Biosciences).

### Extraction and Purification of Gangliosides

Gangliosides were prepared from tumor cells and/or tumor xenografts according to Bouchon et *al.* [18]. Gangliosides were extracted from tumor cells at room temperature with 20 volumes each of 1∶2 and 2∶1 (v/v) chloroform/methanol mixture. The total lipid extract was evaporated to dryness in a rotary evaporator, re-suspended and Folch-partitioned 3 times. The gangliosides were then obtained from the pooled upper phase from the Folch partitions by reverse-phase chromatography on styrene divinylbenzene copolymer columns (EnviChrom™, Supelco, L’isle D’Abeau, France) as described by Popa et *al.* [19]. In some experiments, the O-acetyl groups from gangliosides were removed by alkaline treatment with concentrated ammonium hydroxide overnight at 25°C. The total ganglioside extracts were analyzed by TLC on silica 60 high performance TLC plates (Machery-Nagel GmbH & Co KG, Düren, Germany), using chloroforme/methanol/0.2% aqueous calcium chloride (55∶45:10, by volume). Orcinol reagent was used to visualize the separated gangliosides. Gangliosides were identified by the nomenclature of Svennerholm [20].

### Enzyme Immunostaining on TLC Plates

Detection of GD2 and OAcGD2 was performed by TLC-immunostaining (I-TLC) with mAb ch14.18 and mAb c.8B6 respectively as described by Cerato et *al.* [11]. Briefly, the developed TLC plates were fixed by 0.05% polyisobutylmethacrylate in hexane and treated with PBS-1% BSA for 1 h at 25°C. They were then overlaid with either antibody ch14.18 or c.8B6 at 10 µg/mL in PBS-0.1% BSA overnight at 4°C. After three washings with PBS, mAb binding was detected by stepwise incubation with biotinylated chain specific anti-human immunoglobulins (1∶2000, diluted) (Southern Biotech, Birmingham, AL, USA) for 1 h at room temperature, followed by streptavidin-horseradish peroxidase complex (1∶1000, diluted) (Southern Biotech, Birmingham, AL, USA) for 1 h at room temperature. After extensive washings with PBS, the bound peroxydases were visualized with 4-chloro-1-naphtol solution (Sigma Aldrich, St. Louis, MO, USA).

### Purification of Gangliosides from NXS2 Tumor Bearing Mouse Serum

Serum was collected from subcutaneous NXS2 tumor-bearing mice. For this purpose, A/J mice were injected subcutaneously with NXS2 cells (10^6^ cells) in the right flank. Tumor volume and health of the mice were monitored. After grafts became visible, the tumor size was determined three times a week by externally measuring the tumors in two dimensions. Tumor volume was calculated according to the equation: V = (L**×**W^2^)**×**0.5, where L is the length and W the width of the tumor. Mice were euthanized when the tumors reached 1000 mm^3^. Serum samples were taken. Serum-gangliosides were reclaimed by reverse-phase chromatography on styrene divinylbenzene copolymer columns from sera diluted once (V/V) in methanol according to Popa et *al.* [19]. The total ganglioside extracts were analyzed by I-TLC as describe above.

### Immunohistochemistry

Portions of Sprague–Dawley rat sural nerves or NXS2 tumors grown in A/J mice were embedded in Tissue Tek-II O.C.T. (Miles, Naperville, IL), snap frozen in liquid nitrogen, and stored at −80°C. Ten micrometer-sections were cut, fixed in acetone, and stained with chimeric mAbs c.8B6 or ch14.18 for 1 hour. After washing with PBS, mAb binding was detected by stepwise incubation with biotinylated chain specific anti-human immunoglobulins (1∶500, diluted) (Southern Biotech, Birmingham, AL, USA) for 1 hour at room temperature, followed by streptavidin-horseradish peroxidase complex (1∶1000, diluted) (Southern Biotech, Birmingham, AL, USA) for 1 hour at room temperature. After rinsing, the bound antibody was detected with DAB chromogen substrate solution (Dako, Glostrup, Denmark), which was used to produce a brown deposit. Rituximab was used as a negative control. A mAb specific to mouse neural cell adhesion molecule (CD56) purchased from Abbiotec (San Diego, CA, USA) was used as positive control. The concentration 5 µg/mL was selected for the study because it would result in minimal background and maximal detection signal. Slides were counter-stained with hematoxylin before immunocytological evaluation. Staining was graded as positive or negative according to the presence or absence of immunoreactivity, respectively.

### Radioiodination and Immunoreactivity of Radiolabeled Antibodies

Monoclonal antibodies (30 µg in 50 µl PBS) were labeled with iodine-125 (PerkinElmer, Billerica, MA) using the iodogen method and were purified by gel filtration chromatography on a PD10 column (Sephadex G25, GE Healthcare, Uppsala, Sweden). Immunoreactivity was determined by binding assays of 10 ng of either ^125^I-c.8B6 or ^125^I-ch14.18 with serially diluted NXS2 cell suspension concentrations for 2 hours at 4°C. Cell-bound radioactivity was then separated from free antibody by centrifugation through a dibutylphthalate oil cushion in microfuge tubes. Cell pellets and supernatant activities were then separately measured using a gamma counter (Wallac, Finland). The binding data were analyzed with the Equilibrium Expert software [21] according to a one-site equilibrium binding equation and the percentage of binding was determined by measuring the radioactivity bound to cells. For each experiment, non-specific binding evaluated in the presence of an excess of the unlabeled antibody 8B6 (2.0×10^−6^ M), was modelled as linearly dependent on the cell number.

### Determination of Binding Parameters

A competition cell-binding assay was carried out to assess the reactivity of mAb c.8B6 and mAb ch14.18 against their respective target antigens. Two competitive binding assays between ^125^I-mAb and unlabeled mAbs were carried out on NXS2 cells. Antibodies c.8B6- and ch14.18-binding activities against OAcGD2 and GD2, respectively, were determined by homologous competition binding activity. Antibody ch14.18 cross-reactivity against OAcGD2, and binding activity of c.8B6 to GD2, were studied by heterologous competition using ^125^I-labelled c.8B6 and ch14.18, respectively. For homologous competition assays, the radiolabelled antibody (10 ng) in the presence of increasing concentrations of the corresponding unlabeled mAb (1.7×10^−11^ to 1.0×10^−6^ M) was added to NXS2 cells (1×10^6^) under mild shaking. For heterologous competition experiments, ^125^I-mAb c.8B6 or ch14.18 competed with increasing concentrations of unlabeled ch14.18 and c.8B6, respectively. After 2 h at 4°C, cell-bound radioactivity was separated from free antibody by centrifugation through a dibutylphthalate oil cushion in microfuge tubes. Cell pellet and supernatant activities were then separately measured using a gamma counter (Wallac, Finland). Competition binding curves were plotted as uncorrected bound/total (B/T) as a function of competing antibody concentration and were analyzed with the Equilibrium Expert software according to two-site (GD2 and OAcGD2) equilibrium binding for each antibody. All binding data were analysed simultaneously using the software package taking into account the immunoreactive fraction of the labelled antibodies determined as described above. This analysis allowed an estimation of the binding affinities (expressed in terms of dissociation constant, Kd) of both antibodies to GD2 and to OAcGD2, and the number of binding sites per cell, as well as non-specific binding isotherms.

### Complement Dependent Cytotoxicity (CDC)

Aliquots of NXS2 cells (1×10^4^) were incubated with 80 µL of antibody solution at various concentrations for 2 h at 37°C, human serum (20 µl) was added to provide complement. Cytotoxicity was determined within the tumor cell population after addition of the viability probe propidium iodide (PI) using a FACSCalibur flow cytometer (BD Biosciences, San Jose, CA, USA) and CellQuestPro software (BD Biosciences). The percentage of specific lysis was calculated as: 100× (non viable PI^+^ tumor cells)/(non viable PI^+^ tumor cells+viable tumor cells).

### Antibody-Dependent Cell-mediated Cytotoxicity (ADCC)

An ADCC assay was performed as reported previously [22]. Tumor cells were labeled with membrane dye PKH-26 (Sigma Aldrich, St.Louis, MO, USA) according to the manufacturer’s instructions. Aliquots of the labeled cells (1×10^4^ cells/100 µl) and were incubated with 50 µL of antibodies in 96-well microtiter plates. The human NK-92-RFcγIII+ cells were used as effector cells [23]. Fifty µL of NK-92-RFcγIII+ cells at the indicated effector-to-target ratio (E/T) were added to the tumor cells and incubated for 24 hours at 37°C. Cell death within the PKH-26^+^ target cell population was then assessed by the addition of TO-PRO-3 iodide (TP3) (Life Technologies, Grand Island, NY, USA). The double-positive TP3^+^, PKH26^+^ dead target cell population was detected by flow cytometry analysis using a FACSCalibur flow cytometer (BD Biosciences) in conjunction with CellQuestPro software (BD Biosciences). The percentage of specific lysis was calculated as: 100× (non viable double-positive target cells)/(non viable double-positive target cells+viable PKH26^+^ target cells). The lysis of the NK-sensitive mouse T cell lymphoma YAC-1 was used as an indicator of NK-92-RFcγIII+ activity [24].

### Murine Tumor Model

The anti-neuroblastoma efficacy was determined in the murine NXS2 neuroblastoma experimental liver metastasis model previously described by Lode et *al.* [25]. The NXS2 cell line is a syngeneic murine neuroblastoma in A/J mice by Zeng et *al* [26] in the ch14.18 preclinical setting. This study was carried out in strict accordance with the recommendations in the Guide for the Care and Use of Laboratory Animals of the French Department of Agriculture (agreement number 44–278). The protocol was approved by the Committee on the Ethics of Animal Experiments of the Région Pays de la Loire (Permit Number: B4459). All injections were performed under sodium pentobarbital anesthesia, and standard protocols were made to minimize suffering. Female A/J mice (6–8 weeks of age) were obtained from Harlan Sprague-Dawley (Sulzfeld, Germany). Briefly, 2.5×10^5^ tumor cells were inoculated by tail vein in PBS. After 3 days, mice were injected daily for five consecutive days with dose 200 µg or 25 µg of chimeric mAb c.8B6 or ch14.18, total dose at 1000 or 125 µg. Rituximab (200 µg per dose) was used as a negative control. Mice were sacrificed after 28 days post inoculation, and anti-tumor efficacy was evaluated by liver weight of the fresh specimen.

### Allodynia

Experiments using adult (200–300 g) male Sprague–Dawley, PVG (C+) (Harlan Industries, Indianapolis, IN), were approved by the Institutional Animal Care and Use Committee of the University of California, San Diego (Permit Number: S12314). All rats were allowed to recover from shipping for at least 72 hours prior to experiments. Animals were acclimated to the test facility and the testing equipment during 2 sessions prior to the actual experiment. On the test day, animals were placed in individual plastic compartments (26×11×20 cm) with wire mesh floors for 30 min prior to determination of basal 50% probability mechanical withdrawal threshold for each rat. Animals were then lightly anesthetized with isoflurane (about 2%) and injected with antibody or vehicle through the tail vein, using a 30 g needle. Anesthesia was discontinued after injection and the animal placed back in the testing compartment. Rats were awake within 3 min and in most experiments, mechanical withdrawal thresholds were re-measured every hour for the first 5 h after injection of agent. Experiments began between 9 and 10 a.m. Animals were kept two to a cage under a 12/12 hours day/night cycle with food and water available *ad libitum*. Rats were injected with mAb ch14.18 (anti-GD2 antibody) (1 mg/kg; n = 7), an equal amount of mAb c.8B6 (anti-OAcGD2) (n = 9), an increased dose (3 mg/kg; n = 7) of mAb c.8B6 antibody or rituximab (1 mg/kg; n = 7). One mg/kg of mAb ch14.18 antibody produces maximum allodynia in the rat [27]. The person performing the behavorial testing was blinded to the contents of the syringe. Mechanical withdrawal thresholds following the various treatments were measured with a set of von Frey filaments (Stoelting) with exponentially incremental bending forces ranging from 0.41 to 15.1 g. When the animal was quiet and resting on all four paws, a filament was presented perpendicular to the plantar surface of the hind-paw with sufficient force to elicit a slight bend. Filaments, beginning with the 2.0 g filament, were presented in ascending order of stiffness until an abrupt paw withdrawal (escape) or the stiffest (15.10 g) filament in the set was applied. Stimuli were maintained for 6 s. Successive stimuli were separated by several seconds or until the animal was again calm with hind-paws placed flat on the mesh flooring. Testing followed an up–down paradigm [28], i.e., when a response was made, filaments of decreasing strength were applied until the animal no longer responded, at which point filaments were again presented in ascending order. This pattern was repeated for four stimulus presentations after the first withdrawal response. The 50% probability withdrawal threshold was calculated [29]. Animals that did not respond to the stiffest filament, were considered to have thresholds at cut-off (15.1 g). This process was repeated for both the left and right hind-paws at each timepoint, the average value of the two hind-paws was considered to be the animal’s response.

### Statistic Analysis

Group results are illustrated as mean ± standard error of the mean (S.E.M.). Statistical significance of liver weights of experimental groups of mice was determined by two-tailed Student’s *t*-test p>0.05 using GraphPad Prism software. Percent allodynia area under the curve (AUC) was calculated for each animal based on their individual baseline responses using the trapezoidal method. This was then normalized such that 0 = no change from baseline and 100 = maximal allodynia. Statistics were performed on the 50% probability withdrawal threshold and maximal allodynia using ANOVA for repeated measures and Bonferroni’s post hoc test. *p*<0.05 was considered to be significant.

## Results

### Expression of the Mouse/Human Chimeric mAb c.8B6

The chimeric antibody expression vectors were constructed as described in the Material and Methods section and chimeric c.8B6 was expressed in stably transfected CHO-S cells. We purified mAb c.8B6 from culture medium using protein A affinity chromatography. SDS-PAGE under reducing conditions showed that the molecular weights of chimeric light and heavy chains were 25,000 and 50,000, respectively (data not shown). From analysis by non-reducing SDS-PAGE, the molecular weight of chimeric antibodies was ∼150,000 (data not shown). These results indicated that the chimeric light and heavy chains were assembled as correct tetrameric molecules. Endotoxin levels in the purified mAb solutions were found to be 3.08 EU/ml for mAb c.8B6 and 0.11 EU/ml for mAb ch14.18.

### Specificity of mAb c.8B6 against OAcGD2

**Figure 1:**
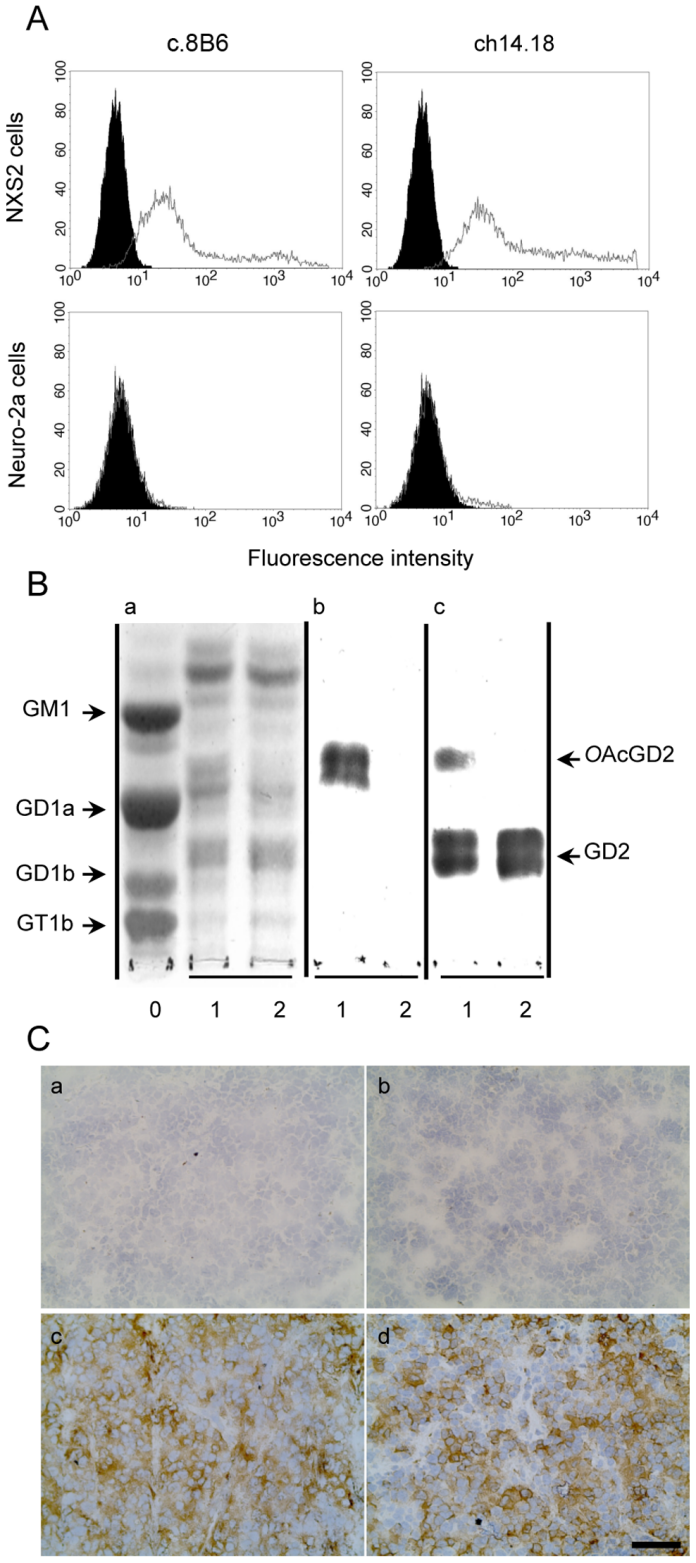
Binding specificity chimeric mAbs c.8B6 and ch14.18 measured by flow-cytometric analysis (A), by TLC-immunostaining (B) and by Immunochemistry on NXS2 tumor (C). (A) NXS2 and the OAcGD2/GD2^−^ Neuro2A cells were stained as described in Materials and Methods. Representative flow cytometry histogram of OAcGD2 expression. Antibody c.8B6 or mAb ch14.18 (*grey*) and control antibody (*black*). The same staining pattern was observed for mAb c.8B6 or ch14.18 on OAcGD2/GD2^+^ NXS2 cells whereas neither mAb c.8B6 nor mAb ch14.18 bind OAcGD2/GD2-negative Neuro-2a cells. These experiments were independently replicated 3 times. (B) TLC of gangliosides extracted from rat brain (Lane 0) and NXS2 cells with (Lane 1) or without (Lane 2) alkaline treatment and stained with resorcinol-HCl (Panel a) or immunostained with mAbs c.8B6 (Panel b), or ch14.18 (Panel c). Chimeric mAb 8B6 reacted with the alkali-labile *O*AcGD2 ganglioside with no cross-reactivity against GD2 ganglioside (Panel b) whereas ch14.18 reacted with GD2 and OAcGD2 (Panel c). The same results were obtained in 3 independent experiments. (C) An immunoperoxidase assay was performed on NX2 neuroblastoma tumor sections as described in Material and Methods. Strong immunostaining was detected on neuroblastoma cells with either mAb c.8B6 (c) or mAb ch14.18 (d). The anti-CD20 chimeric antibody ritximab was used as a negative control (b). No antibody (a). NXS2 neuroblastoma tumors from 6 different mice were tested with the same results. Scale bar = 20 µm.

We reported the specificity of mouse mAb 8B6 for OAcGD2 previously [14]. Here, we studied the specific surface binding of chimeric mAbs c.8B6 for OAcGD2. The binding activity of mAb c.8B6 against the mouse neuroblastoma OAcGD2-expressing NXS2 cells was demonstrated using an indirect immunofluorescence assay and flow cytometry analysis as described in the Material and Methods section. As shown in Fig. 1A, c.8B6 mAb stained virtually all NXS2 cells similar to ch14.18 mAb, whereas no staining was observed with the isotype-matched irrelevant antibody Rituximab. We also observed similar results when we studied the binding activity of mAb c.8B6 on NXS2 tumors using an immunoperoxydase assay. As depicted in Fig. 1C, either mAb c.8B6 or ch14.18 strongly stained NXS2 tumor sections, respectively. The IgG1 isotype control antibody was also negative (Fig. 1C).

We also confirmed c.8B6 mAb specificity for OAcGD2 by I-TLC analysis. Analysis of the ganglioside composition revealed that NXS2 cells contained at least 7 ganglioside species (Fig. 1B, Panel a, Lane 1). After base treatment, the intensity of the ganglioside specie migrating between GD1a and GM1 diminished (Lane 2). This ganglioside species was identified as OAcGD2 by mAb c.8B6 (Pannel b, Lane 1). Its activity against OAcGD2 was lost after alkaline treatment (Panel b, Lane 2) due to the O-acetyl function hydrolysis during alkaline treatment. Importantly, mAb c.8B6 stained exclusively OAcGD2 when tested on total NXS2 neuroblastoma gangliosides (Panel b, Lane 1). In particular, no cross-reaction with GD2 ganglioside was detected before (Panel b, Lane 1) or after (Panel b, Lane 2) alkaline treatment. In the same experiment, we examined the binding activity of mAb ch14.18. Fig. 1B shows that mAb ch14.18 bound specifically to GD2 and cross-reacted to OAcGD2 (Panel c, Lane 1). After alkaline treatment no binding activity to OAcGD2 was observed (Panel c, Lane 2). Taken together, these results demonstrate that chimeric mAb 8B6 retained the same specificity to OAcGD2 as the mouse parental mAb 8B6 with no cross-reactivity to GD2 ganglioside.

### Determination of Binding Parameters

**Figure 2:**
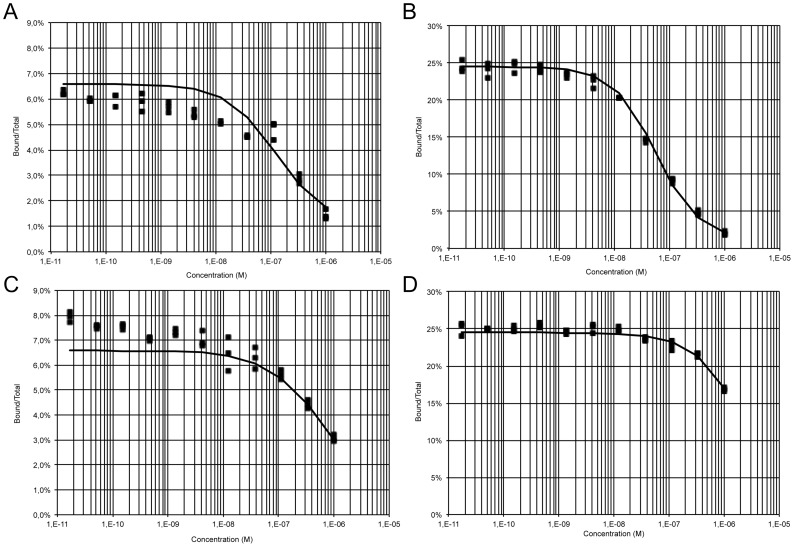
Binding curves of mAb c.8B6 and mAb ch14.18. Iodine-125-labeled mAbs (c.8B6 and ch14.18) were competed with unlabeled mAbs for binding to *O*AcGD2- and GD2-positive NXS2 cells for 2 h at 4°C. Cell-bound radioactivity was separated from free antibody by centrifugation through a dibutylphthalate oil cushion in triplicates and counted. Competition binding curves were plotted as bound/total (B/T) as a function of competing antibody concentration (solid squares). Binding curves were simulated with the Equilibrium Expert software (solid lines). (A), iodine-125-labeled c.8B6 mAb was competed with mAb c.8B6 (A); (B), iodine-125-labeled ch 14.18 mAb was competed with mAb ch 14.18; (C) iodine-125-labeled c.8B6 mAb was competed with mAb ch 14.18; (D), iodine-125-labeled ch 14.18 mAb was competed with mAb c.8B6. Estimated binding parameters are given in Table 1.

A Kd value of 32 nM has previously been reported for mouse mAb 8B6 to OAcGD2 [14]. Here, we delineated the binding affinity (Kd) of both chimeric mAbs c.8B6 and ch14.18 against their respective epitopes using ^125^I-labeled chimeric mAbs c.8B6 and ch14.18. In our radiolabeling antibody experiments, ^125^I-labeled mAb c.8B6 was found to be 48.8% immunoreactive, whereas ^125^I-labeled mAb ch14.18 exhibited 51.3% immunoreactivity (Table 1). The non-specific binding was less than 0.8% (data not shown). Simultaneous analysis of homologous and heterologous competitive binding data using NXS2 cells (Fig. 2) allowed us to obtain an estimate of the Kd values of both antibodies for GD2 and OAcGD2, as well as of the expression of GD2 and OAcGD2 molecules at the surface of NXS2 cells (Table 1). Such a simultaneous analysis of binding data obtained under a variety of conditions is made possible by the Equilibrium Expert software [21] which runs as an add-in to Microsoft® Excel. A partition function, involving the concentrations of the different free molecular species and of the different complexes present in the experiment, is used to describe the experimental system. Binding parameters are estimated by non-linear least square fitting of experimental measurements with user-defined weighting of the experimental data. As shown in Table 1, the estimate of the average number of OAcGD2 binding sites was 0.5±0.2×10^6^ molecules per NXS2 cell whereas the average number of GD2 binding sites was 1.0±0.1×10^6^ per NXS2 in accordance with results obtained with other techniques. The Kd value of mAb c.8B6 for OAcGD2 was estimated as 87.7±28.7 nM (Table 1), and the Kd values of mAb ch14.18 for GD2 and OAcGD2 were 22.6±2.2 and 626±180 nM, respectively (Table 1). Curve fitting for the competition of labeled ch14.18 by unlabeled c.8B6 was improved by assuming that c.8B6 has a weak affinity (Kd >1 µM) for GD2 (Fig. 2D).

**Table 1:** Binding properties of mAbs c.8B6 and ch14.18 on NXS2 cells*.

Antibody	Immunoreactivity(%)	Kd (nM)		Binding site (× 10^6^)	
		OAcGD2	GD2	OAcGD2	GD2
c.8B6	48.8	87.7±28.7	>1000	0.5±0.2	–
ch14.18	51.3	22.6±2.2	626±180	–	1.0±0.1

*Determined as described in the Material and Methods Section. Data are presented as the mean ± SEM for three independent experiments, each in triplicate.

### Chimeric mAb c.8B6 Induces CDC and ADCC in vitro against OAcGD2-expressing Neuroblastoma Cells

**Figure 3:**
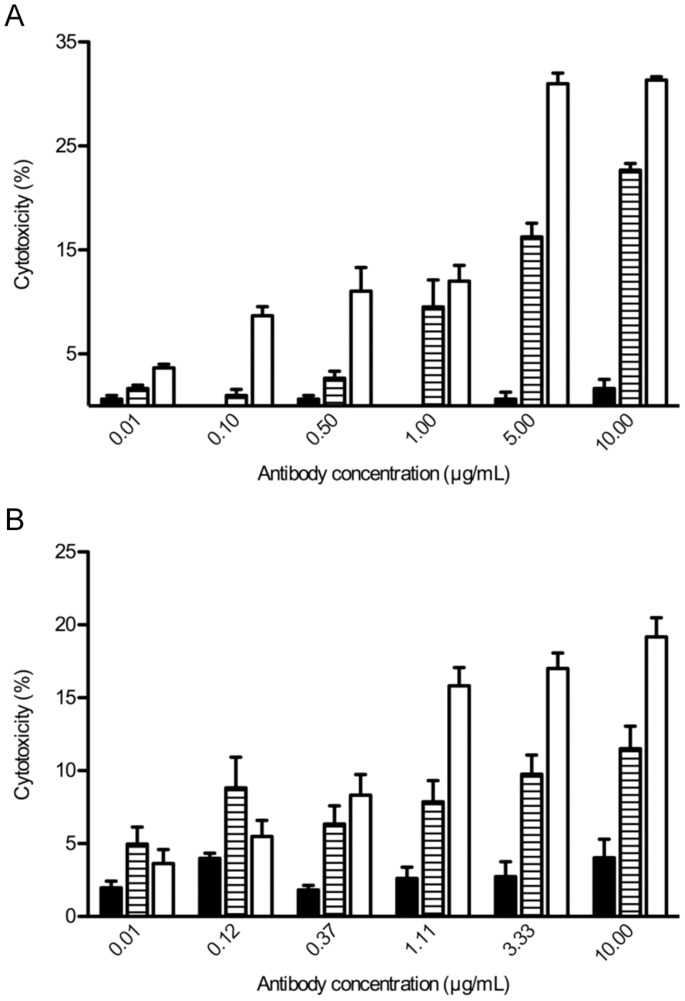
Immune effector functions of both mAbs c.8B6 and ch14.18. (A) Complement-dependent specific lysis was determined for the NXS2 cells as described in Materials and Methods. (B) The NK-92-RFcγIII+ ADCC activity with NXS2 target cells at E/T ratio 12 to 1 as described in Materials and Methods. Horizontally hatched columns, mAb c.8B6; white columns, mAb ch14.18; black columns, irrelevant antibody. Data are presented as the mean ± SEM for three independent experiments, each in triplicate.

In a previous work we described the capacity of the parental mouse mAb 8B6 to induce CDC and ADCC against NXS2 cells [14]. Here, we evaluated and compared the capacity of chimeric mAbs c.8B6 and ch14.18 to induce CDC in the presence of human serum and ADCC by NK-92-RFcγIII+. For the CDC assay, NXS2 cells were incubated with various concentrations of mAbs in the presence of human serum as a source of complement. Cell death was assessed by the addition of the viability probe propidium iodide. CDC was observed with either chimeric mAb c.8B6 or mAb ch14.18 (Fig. 3A). Cytotoxicity was correlated with the concentration of antibody. Specific lysis achieved maximum values of 31.3±0.3% with chimeric mAb ch14.18 and 22.7±0.7% with chimeric mAb c.8B6, at 10 µg/mL. The differences in CDC between mAbs c.8B6 and ch14.18 were statistically significant (p<0.01). We next calculated the EC_50_ of both mAb c.8B6 and ch14.18. The EC_50_ value for c.8B6 was 0.65±0.04 µg/ml and the EC_50_ value of mAb ch14.18 was 0.85 µg/mL ±0.04 µg/ml (data not shown). These data suggest that mAb c.8B6 had similar potency as mAb ch14.18. Specific lysis was demonstrated by comparing the CDC results of mAbs c.8B6 and ch14.18 with the non-specific IgG1 control using Rituximab, which showed only background lysis (Fig. 3A).

For the ADCC assay, NXS2 cells were labeled with the PKH-26 membrane dye, and incubated with various concentration of either chimeric mAbs c.8B6 or ch14.18. The NK-92-RFcγIII+ cells were used as effector cells at 12 to 1 effector-to-target ratio. After incubation, cell death within the PKH-26+ target cell population was detected by the addition of the viability probe TP3. As shown in Fig. 3B, ADCC was observed with either chimeric mAb c.8B6 or ch14.18 on the NXS2 cells. Specific lysis achieved maximum values of 19.2±1.3% for mAb ch14.18 and 11.5±1.5% for chimeric mAb c.8B6. The differences in ADCC between mAbs c.8B6 and ch14.18 were statistically significant (p<0.01). The ADCC activity of mAb c.8B6 was 2.8-fold weaker than mAb ch14.18 when their EC_50_ values were compared, with an EC_50_ value for mAb ch14.18 of 0.64 µg/ml, and an EC_50_ value for mAb c.8B6 of 1.81 µg/ml (data not shown). Maximum capacity of lysis by NK-92-RFcγIII+ was determined to be 64.7±4.8% with the NK-sensitive mouse T cell lymphoma YAC-1 as a target cell (data not shown). Specificity was demonstrated by comparing the ADCC results of either mAb c.8B6 and ch14.18 with the isotype-match irrelevant mAb Rituximab, which showed only background lysis (Fig. 3B). These results suggest that mAb ch14.18 had higher potencies *in vitro* than did mAb c.8B6.

### Chimeric Antibody c.8B6 Demonstrates Anti-neuroblastoma Activity in vivo

**Figure 4:**
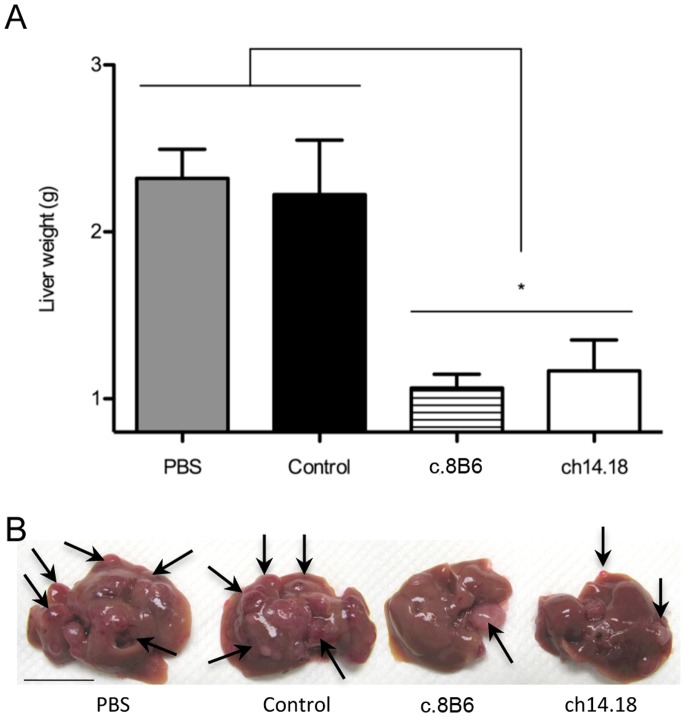
Anti-neuroblastoma activitiy of mAb c.8B6 against established experimental liver metastasis. Mice (n = 6/group) were inoculated with 1×10^5^ NXS2 cells by IV injections. Treatment was initiated three days after tumor cells inoculation and consisted of 5 daily IV injections of 25 µg of mAb c.8B6, mAb ch14.18, and anti-CD20 antibody. Mice were euthanised 28 days post-tumor cell inoculation. (A) The liver weight was determined on fresh specimen. The y-axis starts at 0.8 g corresponding to the average normal liver weight. Data are presented as the mean ± SEM. The differences in mean liver weight between experimental groups treated with mAbs c.8B6 and ch14.18 and all control groups (PBS, control antibody) was statistically significant (*p<0.05). (B) Representative liver specimen of each experimental group is shown. Arrows indicate the location of macroscopic liver metastasis. (Scale bar = 1 cm).

We next determined the anti-tumor efficacy of chimeric mAb c.8B6 using the NXS2 mouse neuroblastoma experimental liver metastasis model [25], and compared its effects to that obtained with mAb ch14.18. This model was previously used by Zeng *et al*. for the preclinical evaluation of mAb ch14.18 [26]. We found that the treatment of mice (n = 6) at the dose of 25 µg of either mAb c.8B6 or mAb ch14.18/day for 5 days was effective in reducing neuroblastoma liver metastasis, as indicated by decreased liver weight from from 2.3±0.2 g (PBS) to 1.1±0.1 g (mAb c.8B6 treated mice) and 1.2±0.2 g for ch14.18 (p<0.05) (Fig. 4A and 4B). The effect of c.8B6 treatment was not statistically different from that obtained upon treatment with ch14.18 (p = 0.94). The specificity of mAb c.8B6 therapy was demonstrated, since treatment with an equivalent amount of the anti-CD20 isotype-matched control antibody Rituximab was completely ineffective. The liver weight in the latter group was found to be 2.2±0.3 g (Fig. 4A).

### OAcGD2 Ganglioside is not Present in the Serum of NXS2 Neuroblastoma Tumor-bearing Mice

**Figure 5:**
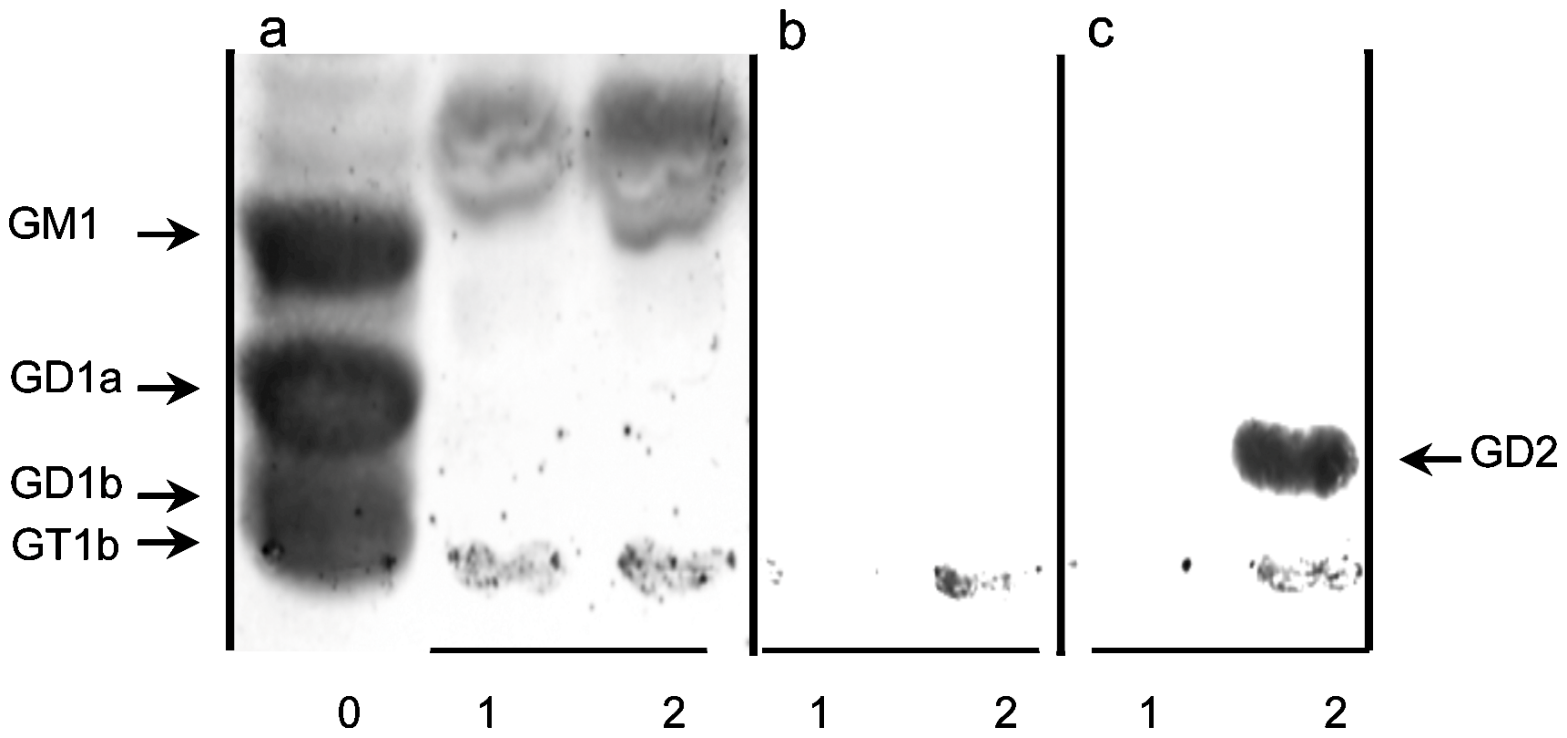
Absence of mAb 8B6-specific OAcGD2 in mouse neuroblastoma sera. I-TLC of gangliosides from rat brain (Lane 0), NXS2 neuroblastoma tumor bearing mice sera (Lane 2), and mouse control sera (Lane 1) stained with resorcinol-HCl (Panel A) or immunostained with chimeric mAbs c.8B6 (Panel B), or ch14.18 (Panel C). Total ganglioside fractions presented in lanes 2 and 3 represents 1 ml of serum. Ganglioside GD2 was detected in the neuroblastoma-tumor bearing mice but not in the control sera when mAb ch14.18 was used. In contrast, no OAcGD2 was detected in sera from neuroblastoma-tumor bearing mice and control mouse sera when either mAb c.8B6 or ch14.18 was tested. Serums from 6 different mice were tested with the same results.

Patients with neuroblastoma were found to have significantly elevated free GD2 levels in serum compared with normal children [30]. Therefore we determined whether the OAcGD2 antigen could be detected in the circulation by analyzing the reactivity of mAb c.8B6 with gangliosides extracted from NXS2 tumor-bearing mice. Gangliosides extracted from 1 ml of serum were separated on a TLC plate and immunostained to assess the binding using an immunoperoxidase assay. Fig. 5 clearly depicts that OAcGD2 is absent in the sera of mouse bearing NXS2 neuroblastoma tumors (Panel b, Lane 2), whereas mAb ch14.18 reacts strongly with GD2 present in the serum (Panel c, Lane 2). Neither OAcGD2 nor GD2 could be detected in normal mouse serum samples (Panel b and c, Lane 1). These data suggest that either OAcGD2 is not shed *in vivo* or that after shedding it might be deacetylated by esterase activity in the serum to yield GD2.

### Chimeric Antibody c.8B6 to OAcGD2 does not Induce Allodynia in Contrast to Anti-GD2 mAb ch14.18

Intravenous infusion of the anti-GD2 antibody in patients is frequently associated with rapid onset spontaneous pain and mechanical allodynia [5], [31]; bolus injection in rats causes quantifiable allodynia within 30 minutes [27]. Location of GD2 ganglioside in peripheral nerves in combination with the ability of anti-GD2 antibodies to induce complement activation and ectopic firing of C ‘pain’ fibers are contributing factors to anti-GD2 antibody induced-allodynia [9], [32]. Therefore, we next compared allodynia evoked by mAbs c.8B6 and ch14.18. First, we analysed mAb c.8B6 reactivity against rat sural nerves by immunohistochemistry using an immunoperoxidase assay. The absence of OAcGD2 expression on human peripheral nerves was reported previously [14]. Figure S1 shows that mAb 8B6 does not bind to rat peripheral nerves (Panel c) whereas mAb ch14.18 reacts with some of the nerves fibers (d). No staining was observed with the control chimeric mAb Rituximab (Panel a). We also used a mAb specific to CD56 mAb as a positive control (Pannel e).

**Figure 6:**
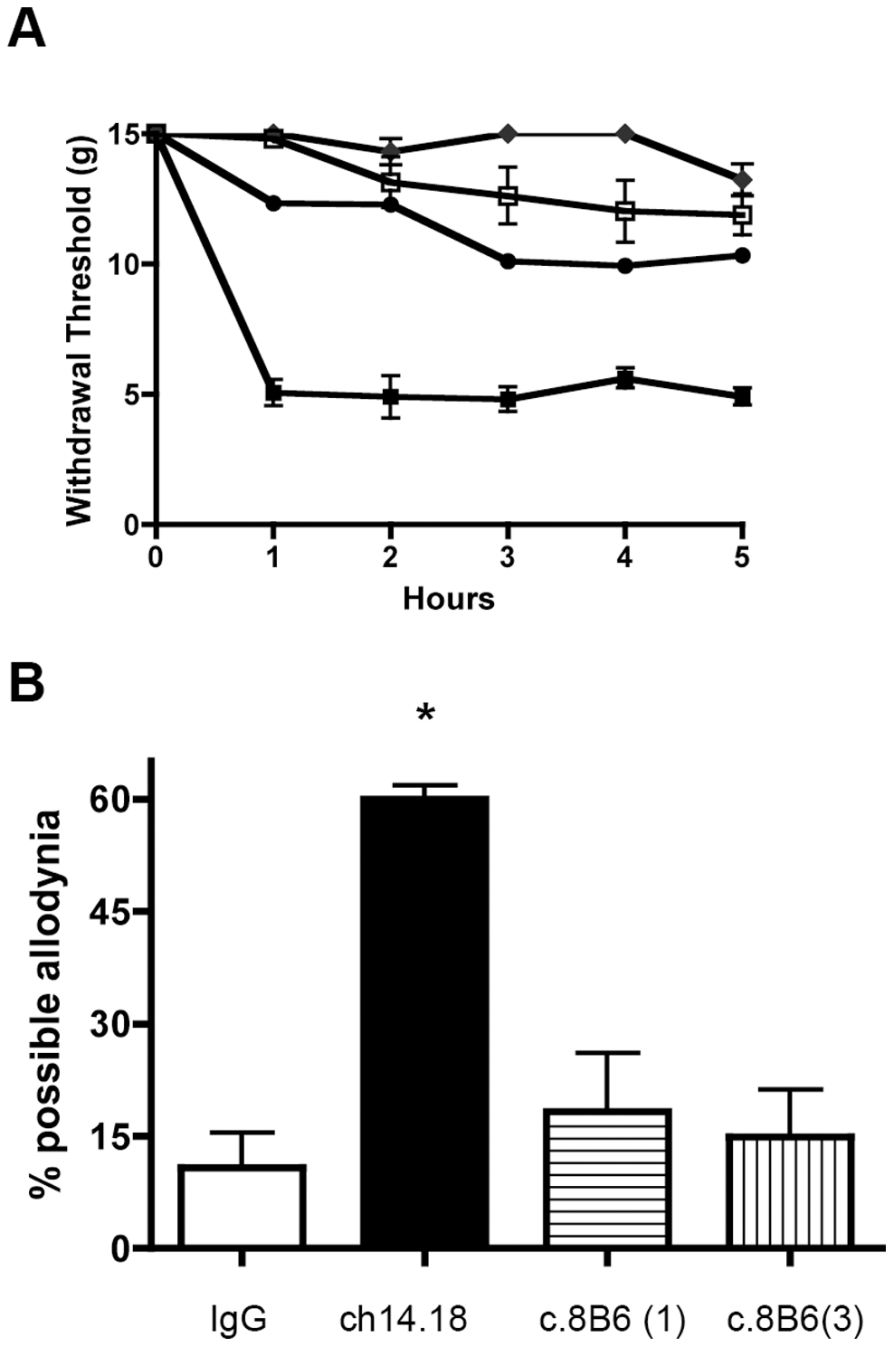
Intravenous injection of mAb c.8B6 does not induce allodynia. (A) Intraveinous injection of either 1 mg/kg (•) or 3 mg/kg (♦)of mAb c.8B6 in Sprague-Dawley rats did not result in a decrease in mechanical thresholds. In contrast, ch14.18 injected at 1 mg/kg (▪) resulted in a prolonged decrease in threshold. Threshold of animal injected with anti-CD20 chimeric control antibody rituximab (1 mg/kg) (□) are shown for comparison. (B) Results shown in panel B were recalculated as area under the curve and then normalized such that 100 would be equal to a theoretical maximal allodynia. Animals administered ch14.18 displayed more allodynia than any of the other treatment groups (p≤ 0.0001).

After demonstrating that mAb c.8B6 did not bind peripheral nerves in rats, we compared mAb c.8B6 to ch14.18 *in vivo* by injecting one or the other antibody into the rat tail vein and testing mechanical allodynia (Fig. 6A). Prior to injection, rats in all groups had 50% probability withdrawal thresholds at cut-off (15.1 g). After IV injection of IgG1-isotype matched irrelevant control antibody, thresholds were essentially unchanged for the next 5 hours and percent allodynia averaged 10.83 g ±4.6, this modest decrease in withdrawal threshold probably results from peripheral sensitization due to repeated testing within a relatively short period of time. Injection of mAb ch14.18 (1 mg/kg) caused thresholds to fall precipitously (Fig. 6B). One hour post-injection, mean mechanical threshold was 4.92 g ±0.4 and remained low, between 3.8 and 5.2 g, for the next 4 hours. Mean percent allodynia for this group was 60.0±1.8 (Fig. 6B). These results duplicated those reported in earlier studies [9], [27]. In contrast, animals treated with either 1 or 3 mg/kg of c.8B6 did not develop allodynia, mean withdrawal thresholds for the animals given the higher dose of c.8B6 were never below 11.5 g for the entire duration of the experiment (Fig. 6A). After mAb c.8B6 injection, percent allodynia was no different that seen in IgG1 control antibody- groups (Fig. 6B).

## Discussion

Anti-GD2 antibody is a proven therapy for GD2-postive neuroblastoma and mAb against GD2 and their derivatives, such as chimeric mAb ch14.18, have provided benchmarks for improving anti-GD2 therapy [3]. Pain can, however, hinder immunotherapy with anti-GD2 therapeutic antibodies such as ch14.18 [3]. The adverse effects are attributed to inflammatory effects via complement activation on GD2-expressing nerves [8], [9]. Consideration for these factors impedes the development of treatment intensification strategies to improve the outcome of neuroblastoma patients. We reported earlier that the O-acetyl derivative of GD2, OAcGD2, is not expressed by human peripheral nerve fibers using the specific mouse mAb 8B6 to OAcGD2. Thus, we suggested that antibodies targeting OAcGD2 offer an effective treatment option with reduced adverse side effects [14]. Human anti-mouse antibody (HAMA) is, however, a limiting factor. Therefore, we established a mouse-human chimeric antibody in order to reduce potential immunogenicity in patients and to fill the need for a selective agent that can kill neuroblastoma cells without inducing neurotoxicity caused by anti-GD2 antibody immunotherapy. We further analyzed some of its functional properties compared with the anti-GD2 ch14.18 therapeutic antibody used in the treatment for children with high-risk neuroblastoma, after stem cell transplant [3].

One criterion for successful chimerization is the preservation of specificity and affinity during genetic engineering. Analogous to its murine counterpart, we showed here that c.8B6 bound to OAcGD2 with reduced or very low cross reactivity against GD2 with an equivalent Kd value [14]. This Kd value is within the range of other anti-ganglioside antibodies reported by others [33], [34], [35]. We also demonstrated that ch14.18 antibody retained the same specificity as its parental mouse antibody against GD2 [12]. We further calculated ch14.18’s affinity for the OAcGD2 epitope, which has not yet been reported, using a cell-binding competition assay. Antibody ch14.18 and c.8B6 compete with each other for binding to the GD2 and OAcGD2 antigen expressed at the cell surface, and ch14.18 showed an apparent approximately 9-fold weaker binding affinity than c.8B6. Moreover, these data allowed us to demonstrate that the number of OAcGD2 molecules present at the tumor cell surface, although 50% of GD2 binding site, is within the range of that of the mAb ch14.18 epitope. These results are in agreement with our previous report [14].

Ch14.18 and other anti-GD2 antibodies have been shown to induce ADCC as well as CDC in GD2-expressing cell lines. A number of preclinical studies have, however, shown that ADCC, is a major mechanism of action of ch14.18 mAb against GD2-expressing cell lines [26]. ADCC is associated with the Fc region of the antibody, and Fc/FcγR interaction is believed to be the mechanism for ADCC. The ADCC effects have been correlated with FcγR interaction affinity for a number of antibodies. Furthermore, the importance of ADCC has also been shown by clinical trials, which have provided evidence of significant correlation between FcγRIIIa functional polymorphisms and clinical outcomes, demonstrated by multiple therapeutic antibodies, including rituximab [36], trastuzumab [37], cetuximab [38], and infliximab [39]. Thus, ADCC is now considered an important clinical mechanism, and enhancing ADCC has become a logical approach to improve the efficacy of anti-GD2 therapeutic antibodies [9] [40]. There is significant variation in the affinities of IgG isotypes for individual Fc receptors, which is reflected by the capacity of active isotypes to recruit immune effector cells efficiently based on their Fcγ receptor expression profile. Fragment Fc-related functions also included long serum half-lives through interaction with the neonatal Fc receptor (FcRn). IgG1 is considered the principal active human isotype based on its comparative affinities for activating receptors [41] and FcRn [42]. Thus, we developed the chimeric c.8B6 with a human IgG1 isotype and demonstrated that anti-OAcGD2 c.8B6 antibody seems particularly effective as compared to ch14.18 therapeutic antibody. Nonetheless, we did observe a 2.8-fold higher ADCC potency of ch14.18. This may due to its binding to both GD2 and OAcGD2, while c.8B6 only binds to the latter. The ch14.18′s ADCC activity against NXS2 neuroblastoma cells reported here is, however, weaker than that reported earlier by Zeng *et al.* [26]. These differences may be related to the chromium-51 release assay used in their study to quantify ch14.18′s ADCC properties. Here we used the flow cytometry-based assay developed by Wilkinson *et al.* [22] because of the alkaline pH of the sodium chromate solution that remove the O-acetyl function of OAcGD2. Thus, this assay may be less sensitive than the chromium-51 release assay.

The use of GD2 antibody in the treatment of cancers also leads to complement dependent cytotoxicity (CDC) [43]. The relationship between complement activation and antibody anti-tumor activity is also suggested for a number of anti-tumor therapeutic antibodies. One of the most convincing examples of the therapeutic importance of this activity is the good clinical responses seen in a phase I study of ofatumumab [44], a second-generation anti-CD20 antibody, which is capable of inducing much more potent CDC activity than rituximab due to its distinct epitope [45]. As mentioned above, all anti-GD2 antibodies mediate efficient CDC [43], and our data suggest that anti-OAcGD2 c.8B6 antibody induces significant, but 4-fold weaker CDC, as compared to ch14.18 therapeutic antibody. This difference may be linked to the lower c.8B6 target site number expressed by the tumor cell as compared to ch14.18 antibody. Yet, anti-GD2 antibody CDC activity is believed to be responsible for the pain side effects [9]. Thus, leveraging ADCC over CDC is one strategy currently used to further optimize the potential clinical efficacy of anti-GD2 antibodies [9], [46]. As an example, a new version of the ch14.18 antibody, hu-14.18K322A, has been made. Hu14.18K332A is a humanized ch14.18 with a single point mutation to alanine at lysine 322 that limits its ability to activate the complement cascade and thereby reduces the pain associated with ch14.18 while retaining its ADCC capabilities. Preclinical studies in rats confirmed that hu14.18K322 elicited substantially less allodynia than ch14.18 [9]. The impact of reduced CDC activity on its anti-tumor potency, however, awaits further clinical investigations. Given these considerations, an overdrive of CDC is probably desirable to further enhance anti-OAcGD2 mAb anti-tumor activity.

We also evidenced earlier that the parental mouse mAb 8B6 induce apoptosis as well [14], [47]. Apoptosis induced by anti-OAcGD2 mAbs is another mechanism that may contribute to their antitumor effects [48]. We were, however, unable to detect apoptosis after tumor cells treatment with either ch14.18 or c.8B6 mAbs in our experiments. The loss of pro-apoptotic activity upon chimerization of mouse mAb 60C3 specific for GD2 ganglioside was also observed previously [17]. Although the cell death mechanism initiated by the mouse mAb 8B6 have not been yet elucidated, our results suggest that the pro-apoptotic activity of anti-GD2 and anti-OAcGD2 mAbs can not fully explained by their sole antigen recognition activity. This question is currently under investigation in our laboratory.

The main purpose of our study was to compare the *in vivo* anti-tumor effect of chimeric c.8B6 to ch14.18 therapeutic antibody on neuroblastoma tumors. Therefore we evaluated the anti-neuroblastoma activity of c.8B6 in the syngeneic NXS2 mouse neuroblastoma in A/J mice as used previously in the preclinical setting of mAb ch14.18 by Zeng *et al*. [26]. Despite a weaker anti-tumor activity measured *in vitro*, we demonstrated here that c.8B6 was able to inhibit NXS2 liver metastasis as efficiently as the ch14.18 therapeutic. More importantly, our *in vivo* studies in rat showed that intravenous c.8B6 treatment did not induce allodynia as compared to ch14.18. Our results confirm earlier findings demonstrating profound mechanical allodynia resulting from intravenous ch14.18 treatment due to peripheral nerve cross-reactivity [9]. Our principal novel finding here is that anti-OAcGD2 mAb c.8B6 did not induce allodynia, even at the dose of 3 mg/kg. The mechanical withdrawal threshold values observed in the rats receiving mAb c.8B6 infusion was comparable to that observed with anti-CD20 rituximab mAb used as a non-specific antibody. These data were consistent with to the absence of c.8B6 mAb binding activity with rat peripheral nerves, and argue that antibodies targeting OAcGD2 may offer an effective option for the development of treatment intensification strategies.

Studies by others demonstrated that GD2 is circulating in the sera of neuroblastoma patients [30]. Schulz *et al*. demonstrated further that GD2 serum levels inhibited anti-GD2 mAb binding to the corresponding neuroblastoma targets *in vitro* [30]. Our data demonstrate the absence of circulation OAcGD2 in the mice sera and suggest that OAcGD2 is either not shed *in vivo* or else is deacetylated by ample esterase activity in the serum to yield GD2 after shedding. The clinical relevance of these findings on anti-GD2 mAb pharmacokinetics properties, however, needs to be investigated.

Thus, c.8B6 antibody is a novel agent that possesses the same anti-neuroblastoma attributes as therapeutic anti-GD2 mAb when tested in cell-based assay and *in vivo* in an animal model. The absence of OAcGD2 expression of nerve fibers and the lack of allodynia–which are believed to play a major role in mediating ant-GD2 mAb dose-limiting side effects–provide an important rationale for the *in vivo* application of c.8B6 in patients with high-grade neuroblastoma.

## Supporting Information

Figure S1 **An immunoperoxydase assay was performed as described in Material and Methods on sural rat nerves.** Antibody c.8B6 did not react with nerve fibers (b) whereas myelin sheaths were stained with mAb ch14.18 (c). The anti-CD20 chimeric antibody was used as a negative control (a) and the anti-CD56 mAb as a positive control (d). Scale bar = 50 µm.
(TIF)
