# Correction: Chimeric Antibody c.8B6 to O-Acetyl-GD2 Mediates the Same Efficient Anti-Neuroblastoma Effects as Therapeutic ch14.18 Antibody to GD2 without Antibody Induced Allodynia

**DOI:** 10.1371/journal.pone.0103395

**Published:** 2014-07-17

**Authors:** The PLOS ONE Staff

In Table 1, the Kd values of ch14.18 for OAcGD2 and GD2 are incorrectly switched. The Kd value of OAcGD2 is 626 ± 180 and the Kd value of GD2 is 22.6 ± 2.2.

Please see the correct Table 1 here.

**Table 1:** Binding properties of mAbs c.8B6 and ch14.18 on NXS2 cells*.

Antibody	Immunoreactivity (%)	Kd (nM)		Binding site (x 10^6^)	
		OAcGD2	GD2	OAcGD2	GD2
c.8B6	48.8	87.7 ± 28.7	> 1000	0.5 ± 0.2	-
ch14.18	51.3	626 ± 180	22.6 ± 2.2	-	1.0 ± 0.1

*Determined as described in the Material and Methods Section. Data are presented as the mean ± SEM for three independent experiments, each in triplicate.
