# Supplementary figures and images for: Chimeric Antibody c.8B6 to O-Acetyl-GD2 Mediates the Same Efficient Anti-Neuroblastoma Effects as Therapeutic ch14.18 Antibody to GD2 without Antibody Induced Allodynia
Source: PLoS One. 2014 Feb 10;9(2):e87210. doi: 10.1371/journal.pone.0087210 (PMC3919714; doi:10.1371/journal.pone.0087210)

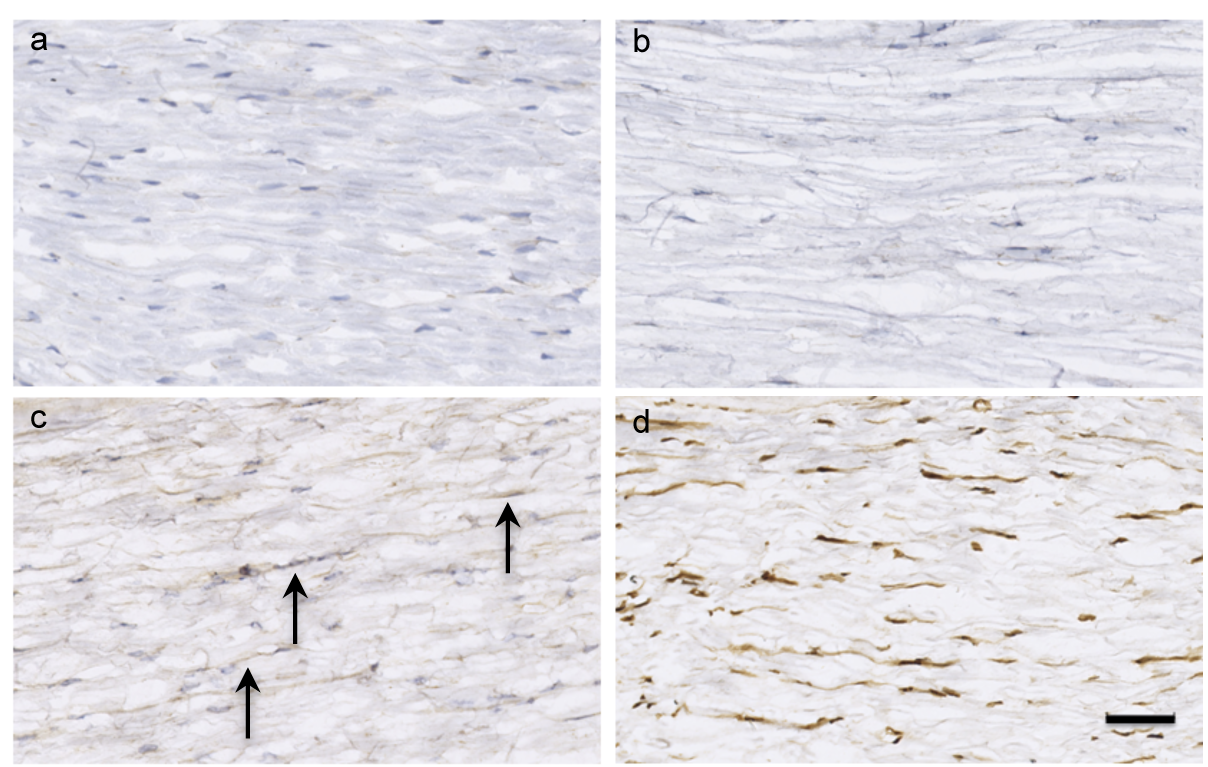

Supplement: Figure S1 — An immunoperoxydase assay was performed as described in Material and Methods on sural rat nerves. Antibody c.8B6 did not react with nerve fibers (b) whereas myelin sheaths were stained with mAb ch14.18 (c). The anti-CD20 chimeric antibody was used as a negative control (a) and the anti-CD56 mAb as a positive control (d). Scale bar = 50 µm. (TIF) [file pone.0087210.s001.tif]
